# Principal components analysis based control of a multi-dof underactuated prosthetic hand

**DOI:** 10.1186/1743-0003-7-16

**Published:** 2010-04-23

**Authors:** Giulia C Matrone, Christian Cipriani, Emanuele L Secco, Giovanni Magenes, Maria Chiara Carrozza

**Affiliations:** 1 Department of Computer Engineering and Systems Science, University of Pavia, Via Ferrata 1, 27100 Pavia, Italy; 2 ARTS Lab, Scuola Superiore Sant'Anna, V.le Piaggio 34, 56025 Pontedera (PI), Italy; 3 EUCENTRE Foundation, Via Ferrata 1, 27100 Pavia, Italy

## Abstract

**Background:**

Functionality, controllability and cosmetics are the key issues to be addressed in order to accomplish a successful functional substitution of the human hand by means of a prosthesis. Not only the prosthesis should duplicate the human hand in shape, functionality, sensorization, perception and sense of body-belonging, but it should also be controlled as the natural one, in the most intuitive and undemanding way. At present, prosthetic hands are controlled by means of non-invasive interfaces based on electromyography (EMG). Driving a multi degrees of freedom (DoF) hand for achieving hand dexterity implies to selectively modulate many different EMG signals in order to make each joint move independently, and this could require significant cognitive effort to the user.

**Methods:**

A Principal Components Analysis (PCA) based algorithm is used to drive a 16 DoFs underactuated prosthetic hand prototype (called CyberHand) with a two dimensional control input, in order to perform the three prehensile forms mostly used in Activities of Daily Living (ADLs). Such Principal Components set has been derived directly from the artificial hand by collecting its sensory data while performing 50 different grasps, and subsequently used for control.

**Results:**

Trials have shown that two independent input signals can be successfully used to control the posture of a real robotic hand and that correct grasps (in terms of involved fingers, stability and posture) may be achieved.

**Conclusions:**

This work demonstrates the effectiveness of a bio-inspired system successfully conjugating the advantages of an underactuated, anthropomorphic hand with a PCA-based control strategy, and opens up promising possibilities for the development of an intuitively controllable hand prosthesis.

## Background

In the last thirty years several examples of robotic hands have been developed by research or industry, some designed to mimic the human hand in its manipulation dexterity and functionality, some aimed at achieving better anthropomorphism and cosmetic appearance [1]. Great research effort has been focused on the design of both articulated articulated end-effectors and smart dexterous anthropomorphic hands, for humanoid robotics and prosthetics. An exhaustive summary of the various approaches and solutions is given in [2] and [1].

An advanced neuro-controlled prosthetic hand bi-directionally interfaced with a human being should address both functional and cosmetic issues; it should be dexterous enough to allow the execution of Activities of Daily Living (ADLs), and include proprioceptive and exteroceptive sensors for the delivery of consciously perceived sensory feedback [3]. Market available myoelectric hand prostheses [4-6] are instead similar to rough pincers [7], having just one (open/close the hand) or two (prono/supinate the wrist) degrees of freedom (DoFs), therefore poor manipulation capabilities. They are controlled by means of electromyographic (EMG) signals picked up from the residual muscles by surface electrodes, amplified and processed to functionally operate the hand [8-10]. Also the recently commercialized multi-fingered I-Limb prosthesis (Touch EMAS Ltd., Edinburgh, UK) [11] is controlled using a traditional two-input EMG scheme where all fingers open/close simultaneously.

The communication interface between the user and the machine is the technological bottle-neck [12] which explains why current hand prostheses are very simple from a biomechanical point of view, even if more sophisticated solutions would be possible. Still nowadays there is no way to easily interface the amputee with the multi-DoF dexterous prostheses developed in the past decades (e.g. the Southampton-REMEDI [13], the RTR II [14], the MANUS [15], the Karlsruhe hands [16], the SmartHand [17], the IOWA hand [18]), since it requires either too many independent control signals or a controller able to compensate for the limited bandwidth of the source signal.

As a matter of fact, increasing the number of DoFs (i.e. dexterity) means either that the system should take care of carrying out the grasp with some level of automatism, as in the SAMS [10,13,19], or that the user should learn how to correctly and selectively modulate different muscular contractions so as to move each prosthesis joint independently (as in [20,21]). In all cases, a certain level of shared-control between the user's intention and the automatic controller is required, as formally introduced by [22]. If the control relies on the automatic controller of the prosthesis, this must include a high number of sensors and intelligent control algorithms to achieve the grasp; on the other hand, if the control system is based on user's intentions decoded from bio-signals extracted by an appropriate interface, (possibly) complex EMG processing algorithms and a high level of training for the user may be required, which could cause fatiguing burden [23]. This could potentially induce the subject to reject the prosthesis, particularly when the amputation is mono-lateral and he/she can supply with the healthy limb to his/her motor deficiency.

An innovative shared-control strategy could be achieved by observing and mimicking the natural biomechanical behaviour. As several studies in the neurophysiology literature report, low-dimensional modules formed by muscles activated in synchrony - also called "muscular synergies" - are used by the human nervous system to build complex motor output patterns during motor tasks [24,25]. In 1997/8 Santello and Soechting reported a series of interesting experimental results on the analysis of human hand grasping postures [26,27], demonstrating that such synergies exist also in hand postural data, which can thus be described in a reduced dimensionality space [26-30].

This concept has been exploited with the aim of controlling robotic grippers and dexterous hands by means of a lower-dimension input space, in a limited number of works. Brown and Asada explored the concept of biomechanical synergies and how they can be applied to a 17 DoFs robot anthropomorphic hand, by mechanically implementing Principal Components Analysis (PCA) and using common patterns of actuation called eigenpostures [31]. Ciocarlie et al. [32] used PCA to design an automatic grasp planning system for integration into the control system of a prosthetic arm and hand driven by cortical activity. Ciocarlie, Goldfeder and Allen [33,34] applied the eigengrasp concept to 5 dexterous hand virtual models (and to a real three-fingered gripper) and derived a grasp planning algorithm. Tsoli and Jenkins [35] compared several different dimensionality reduction techniques used to extract 2D non linear manifolds from human hand motion data and drive the DLR/HIT robotic hand [36]; they also showed how it could be controlled simply using a 2 DoFs input signal like the mouse pointer position [37]. Rossel et al. [38] used the SAH hand [39] and the concept of principal motion directions to reduce the hand workspace dimension.

In the present work a control method based on PCA (preliminary introduced in [40] and [41]) and its implementation in a 16-DoFs underactuated hand (the CyberHand prototype [3]) are presented. The developed strategy allows to achieve a dimension reduction of the control both algorithmically (using PCA) and also mechanically (by means of underactuation). By this way, two independent input signals can be used to drive the hand and to make it grasp different objects representing the prehensile grasping forms mostly used in ADLs. A direct interaction between the user and the robot hand is made possible by combining the user input signals and the matrix which operates the transformation between the input 2D space and the 16-dimensional hand DoFs space. By this way, fingers are somehow directly moved by the user's intention, albeit each single joint position cannot be actively controlled. The final joints configuration is in the end achieved thanks to the hand underactuated mechanism.

The feasibility of exploiting such a control method for achieving real stable grasps is shown here on an anthropomorphic, underactuated prosthesis for the first time. This paper first of all describes the underactuated hand used, the proposed PCA-based control algorithm and particularly how the PCs matrix has been *ad-hoc *built collecting data from the CyberHand sensors, in order to operate dimensionality reduction. The employment of this control strategy in driving the hand during the most typical grasps in ADLs is then presented. Different working conditions have been considered, in order to test the algorithm feasibility both simulating EMG user-generated control signals (more realistic noisy inputs) and in the ideal case. The results obtained performing different grasping trials are finally described and discussed.

## Methods

### The robot hand

**Figure 1:**
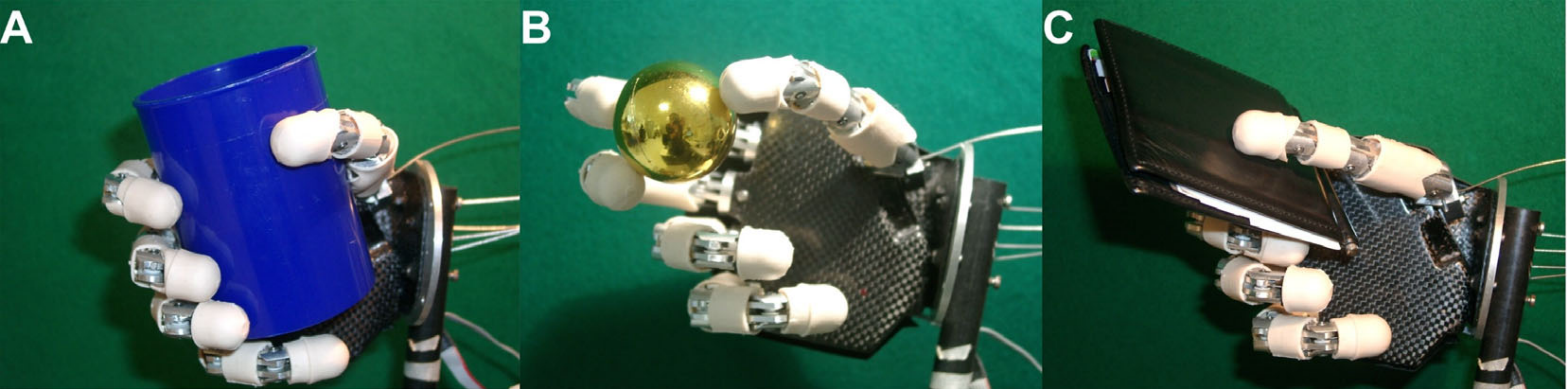
**Power, precision and lateral grasp**. The CyberHand performing the three main grasps as defined by [43]. A) Power grasp: all palmar surfaces of the fingers (as well as the palm) are involved and the thumb is in opposition to other fingers. B) Precision grasp: thumb, index and middle fingertips are involved with the thumb in opposition space. C) Lateral grasps: the thumb opposes to the volar aspect of the index.

The human-sized robot hand used is a stand-alone version of the CyberHand prototype [3]. It consists of five underactuated anthropomorphic fingers based on Hirose's soft finger mechanism [42], which are actuated by six DC motors. Five of them are employed for fingers flexion/extension; thus, each finger has 1 degree of actuation (DoA) and 3 DoFs, since it is composed of three phalanxes. One more motor drives the thumb ab/adduction, which makes a total amount of 16 DoFs [3]. The CyberHand is able to perform the three main functional grasps defined in Iberall's & Arbib's grasp taxonomy [43] and shown in Figure 1: power, precision and side opposition (lateral) grasps.

**Figure 2:**
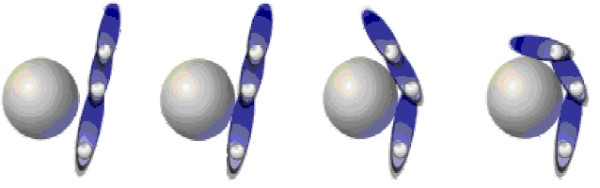
**CyberHand fingers structure**. Conceptual scheme of the underactuated mechanism of the CyberHand finger based on Hirose's soft finger [42].

The fingers of the CyberHand comprise three phalanxes connected by hinge joints and on the hinge axes are assembled idle pulleys. A tendon is wrapped around each pulley from the base to the tip. The tendon is fixated at the fingertip and runs around the idle pulleys in the joints (metacarpophalangeal, MCP; proximal-interphalangeal, PIP; distal-interphalangeal, DIP). When the tendon is pulled, by means of a linear slider actuated by a DC motor, the phalanxes flex starting from the base to the tip. When the motor releases the cable, torsion springs in the joints extend the finger. The CyberHand fingers thus exploit a differential mechanism that is based on elastic elements and mechanical stops. When the finger moves idling (that is, without contacting any object), the kinematics of such an underactuated finger depends on the length of the links/phalanxes, on the radii of the pulleys and on the stiffness of the joint torsion springs. These parameters have been chosen to obtain an anthropomorphic appearance (also while moving) and a stable tip-to-tip pinch based on biological and neuroscience studies [44,45]. In case of object contact, the finger wraps automatically around the object exerting a uniform force: when a phalanx touches the object, thanks to the idle pulleys, the cable can be further pulled, flexing the more distal phalanx (cf. Figure 2). The main drawback of this mechanism is that each finger joint can not be actively and independently controlled.

The hand contains position (encoders integrated in the motors) and tendon tension sensors (able to measure the grasp force [46]), that can be read externally by means of a standard RS232 bus and an implemented communication protocol. The control is embedded in the hand in a 8-bit microcontroller-based hierarchical architecture (Microchip Inc. microcontrollers) and triggered by external commands from the communication bus. According to the serial communication protocol, the set-point positions for each finger are encoded using 8 bits, i.e. from 0 (finger completely extended: all joint angles = 0 deg) to 255 (finger completely flexed: all joint angles = 90 deg).

### PCA-based control algorithm

The PCA algorithm [47] allows to convert an original data set into a new space where dimensions are uncorrelated; it can be briefly summarized as follows. If we suppose to have a (*N *× *M*) dataset matrix, where *N *is the dimension of the original amount of data and *M *is the dimension of each datum, its covariance matrix is a (*M *× *M*) matrix whose eigenvectors are the PCs, and their respective eigenvalues are the PCs weights (i.e. the amount of explained variance). The PCs can then be ordered in descending order according to their weights and used to constitute the columns of the PCs matrix (*M *× *M*). Therefore, by multiplying the original dataset by this matrix, a new (*N *× *M*) dataset is obtained, where rows/data are uncorrelated. Moreover, if the last PCs have a very low weight, they can be neglected (i.e. set to zero), obtaining a new dataset with reduced dimensionality, if compared to the original one.

Consequently, the PCA approach can be used for dimensionality reduction, just inverting its algorithm (explained above) and neglecting the less significant (low weight) PCs [41]. For example, when working with a *M*-DoFs hand and a specific postures data set, we obtain *M *PCs constituting the *M *columns of the PCs matrix, once ordered according to their weight. If we suppose that only the two first PCs are significant, 2 inputs (*In*_*1 *_and *In*_***2***_), which represent the two principal hand DoFs in the new space, can be coupled to the first two PCs and remapped to the hand original *M *DoFs using the PCs matrix obtained from experimental data:(1)

here the output vector consists of the desired *M*-DoFs of the hand. The remaining components of the input vector, which are to be multiplied by the last PCs, are set to zero, in order to neglect the less significant PCs contribution.

This strategy could be exploited with a myoelectric hand prosthesis, where only few signals are available for control, but dexterity is desirable. By employing this "inverse PCA" algorithm, all DoFs of a dexterous robotic hand may be controlled in synergy by means of a simple two-signals control interface, e.g. two independent EMG channels tapped from the residual limb.

In a previous work, this control method had been firstly tested onto a virtual-reality model of a 15 DoFs hand [40]. Simulations of hand movement were performed employing a real human hand PCs matrix available from Santello et al. [26], and the 2-DoFs mouse signal was assumed as the input control signal. The controller received the *x y *real time coordinates of the mouse pointer over the monitor screen, properly calibrated into *In*_*1 *_and *In*_*2 *_range values (found in [26]), and finally, multiplying by Santello's PCs matrix, the virtual hand instantaneous movements were calculated and virtually performed.

Wishing to employ the same control principle to drive a real robotic hand, like the CyberHand, all the described experimental procedure must be reproduced, entirely working with the artificial hand. To this aim, in order to control the six actuators of the CyberHand, a specific PCs matrix has been built just using the CyberHand prototype. The 29 objects listed in Table 1, and reflecting in their different shape and distribution the percentage of different grasps used in ADLs [48], were firmly grasped by the CyberHand and the 6 position values read from motor encoders have been used to constitute each record of the data-set (a (50 × 6) matrix, where 50 is the number of performed trials and *M *= 6 is the dimension of data).

**Table 1:** Grasped objects, used to constitute the CyberHand postures data-set

Object	Shape	Size [mm]	Grasp Type
Paper roll	Cylindrical	Diam = 80; height = 100	Power grasp

Plastic cup	Cylindrical	Diam = 65; height = 90	Power grasp

Small plastic cylinder	Cylindrical	Diam = 36; height = 125	Power grasp

Medium plastic cylinder	Cylindrical	Diam = 41; height = 120	Power grasp

Big plastic cylinder	Cylindrical	Diam = 71; height = 120	Power grasp

Sponge	Cylindrical	Diam = 100; height = 36	Power grasp

Glue bottle	Cylindrical	Diam = 45; height = 130	Power grasp

Spray	Cylindrical	Diam = 50; height = 135	Power grasp

Twine roll 1	Cylindrical	Diam = 106; height = 21	Power grasp

Twine roll 2	Cylindrical	Diam = 40; height = 75	Power grasp

Tennis ball	Spherical	Diam = 65	Power & precision grasp

Plastic sphere 1	Spherical	Diam = 40	Precision grasp

Plastic sphere 2	Spherical	Diam = 49	Precision grasp

Plastic sphere 3	Spherical	Diam = 59	Precision grasp

Fabric ball	Spherical	Diam = 70	Precision grasp

2 liters bottle	Cylindrical	Diam = 90	Power grasp

500 ml bottle	Cylindrical	Diam = 65	Power grasp

Boxes seal tape	Empty cylinder	Diam = 90; height = 50	Power & precision grasp

Felt tip pen	Cylindrical	Diam = 16; height = 130	Precision grasp

Plastic cube	Cube	L = 50	Precision grasp

CD	Circular	Diam = 120	Precision grasp

Electric adapter plug	Cylindrical	Diam = 41	Precision grasp

CDs pack	Cylindrical	Diam = 125; height = 70	Power grasp

Styrofoam sphere	Spherical	Diam = 90	Power & precision grasp

Cigarette pack	Parallelepiped	20 × 55 × 85	Power & lateral grasp

Card box 1	Parallelepiped	103 × 58 × 45	Power grasp

Card box 2	Parallelepiped	103 × 45 × 40	Power grasp

Paperclips pack	Parallelepiped	55 × 39 × 11	Lateral grasp

Business card	Rectangular	Height = 1	Lateral grasp (× 10)

Objects used to collect the data-set from the CyberHand for calculating the PCs matrix. Lateral grasps have been repeated 10 times (in order to obtain the correct percentages values for power, precision and lateral grasps based on [48]), and some objects have been grasped using different hand configurations (i.e. grasping the seal tape with the fingertips rather than leaning it against the hand palm, or holding the sphere with the hand fingertips rather than performing a spherical grasp). The open-hand position has been included into the data-set (4 times).

The obtained new matrix allows to calculate the 6 motor set-point positions (6 elements output vector in eq. (1)). Only the first two PCs have been considered significant (accounting for more than 90% of the data variance) and used subsequently to drive the hand (the remaining four PCs have been multiplied by a zero input).

### Two-inputs control interface

As a proof of concept, two independent signals like the mouse vertical and horizontal position signals have been used to modulate the two first PCs with the aim of demonstrating that they can be employed to achieve significant hand dexterity.

**Figure 3:**
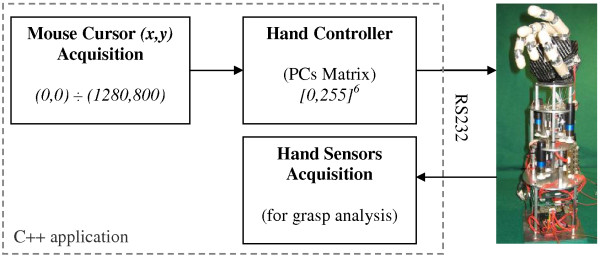
**System block diagram**. Mouse position values are acquired in real time and converted in six 8-bits position control commands for the hand. Artificial sensors in the hand are available for grasp and prehensile capabilities analysis.

In order to experimentally test the potentiality of this control approach onto a real multi-DoF underactuated hand, a C written application for bi-directionally interfacing with the hand was implemented using LabWindows CVI (National Instruments Corp., Austin, TX, USA). The software, running on a standard PC and graphically presented in Figure 3, generates *In*_*1 *_and *In*_*2 *_by acquiring (sampling frequency 100 Hz) the mouse cursor coordinates. It calculates the 6 set-point position values for the hand fingers by multiplying the two inputs for the CyberHand PCs matrix and sends them to the hand by means of the RS232 communication bus. Such program is also used to sample and acquire tendon tension and position sensors data.

### Experimental protocol

**Figure 4:**
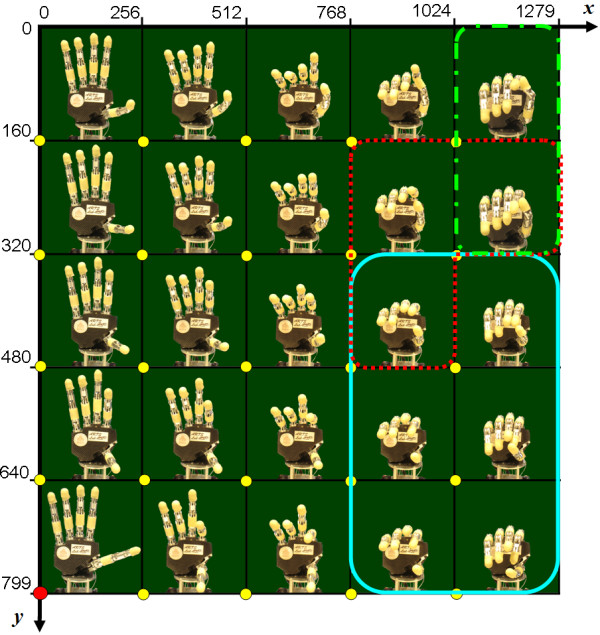
**CyberHand postural behaviour**. A grid representing hand postures distribution over the *xy *screen reference system (monitor screen size is 1280 × 800 pixels, w × h). Circular yellow markers indicate those mouse pointer positions used to drive the hand until the corresponding posture was reached. When the mouse is positioned in correspondence of the red marker, open hand configuration is obtained. The solid, dotted and dashed-dotted lines delimit those areas in which respectively power, precision and lateral grasps can be achieved.

To allow a more immediate interpretation, results in this paper are presented with reference to the *xy *monitor screen plane; this is equivalent to the *In*_*1 *_and *In*_*2 *_plane, since the two spaces are proportionally bounded. Figure 4 shows a discrete *xy *grid and how the hand behaves when varying *In*_*1 *_and *In*_*2*_, i.e. moving the mouse pointer over different areas of the screen using the computed PCs matrix. The map highlights that some areas (i.e. some PCs combinations) are more functional for certain grasp types rather than others. Generally, an excursion along the *x *axis (which is coupled with PC_1_) principally influences fingers flexion/extension, whereas variations along the *y *axis (coupled with PC_2_) mostly influence thumb abduction and slightly make the other fingers flex/extend.

A neutral position area has been established in the left bottom corner of the map. With the mouse cursor in this area (a 15 × 15 pixels square area) the hand opens shaping in a relaxed posture. This option is fundamental for the application under investigation, as a grasp usually starts from the hand being opened. The farthest end area chosen is easily reached with a wide movement of the mouse (or a strong contraction of the residual muscles, considering a myoelectric controller) and does not require a precise positioning (as e.g. with the neutral area in the centre of the screen). Besides, the left bottom corner corresponds to an almost opened hand posture also when using the PCs matrix by itself.

The investigation on prehensile capabilities has been focused on the three forms indicated by Iberall & Arbib [43]. Three control objects have been used: a 500 ml bottle as a prototypical power grasp (dimensions in Table 1), a small sphere for the precision grasp, (cf. Plastic sphere 1 in Table 1) and a credit card for the side opposition/lateral grasp. The experiment consisted in using the mouse for stably grasping the object, starting with the hand in the relaxed-like position. The mouse was moved along linear trajectories and once the grasp was achieved, stable sensor values were collected and the *x, y *pointer coordinates were noted down. Stable grasp points were characterized in terms of:

- number of fingers actually involved in holding the object;

- tendon tension summation, i.e. grasping force [22,46].

**Figure 5:**
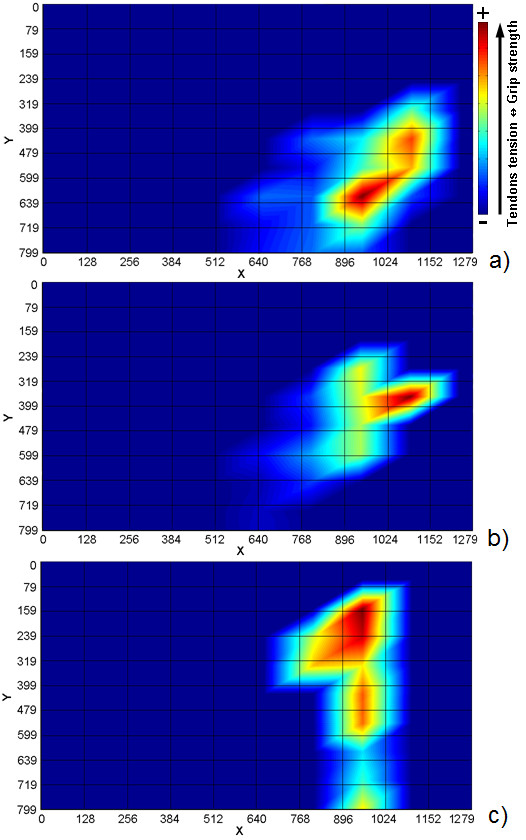
**Grasp type areas**. Color-intensity maps representing the hand total tendons tension (i.e. grasp strength) distribution with respect to the monitor screen reference system, while performing three different grasps: a) power, b) precision and c) lateral grasp. Each map has been built recording tension values and the corresponding mouse *xy *position whenever a stable grasp has been achieved by the mouse-driven hand.

This procedure was manually executed and repeated (for each of the 3 objects/prehensile forms) in order to qualitatively localize *grasp areas *and for these *grasp areas *quantitatively represent the grasping force. Figure 5 shows the three maps obtained on the *xy *reference system, with color intensity based on the tendon tension summation.

The maps in Figure 5 help to approximately evaluate the direction along which grasp strength increases for each grasp type, and how grip force changes when moving along different directions in the neighborhood of stable grasp points. Due to the mechanical configuration of the hand, for what concerns power and lateral postures (partially form-closure grasps [49]), an increase of the tendon tensions summation actually represents an increase in resistance to slipping [22,50]. This is not true for precision grasps, for which high tendon tensions summation values (high strength grasp) could lead to roll-back phenomenon with consequent loss of stability [51].

**Figure 6:**
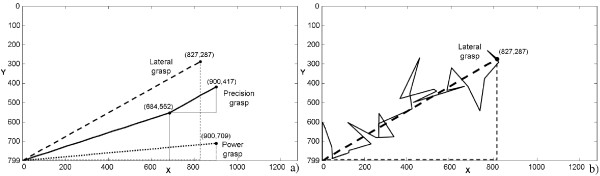
**Pre-calculated grasping trajectories**. Pre-calculated *xy *trajectories used to drive the hand in the 3 different grasping prehensile forms. a) The three ideal linear trajectories (bold lines) and "right-angle" trajectories (thin lines) obtained moving along horizontal and vertical line segments. b) Ideal (bold dashed line), noisy (70 pixels maximum noise amplitude, solid line) and "right-angle" trajectories (thin dashed line) in the lateral grasp case.

The possibility of exploiting the PCA based algorithm for dexterous prosthesis grasp control has been finally investigated as follows. The hand was used to grasp the three objects and was driven by pre-computed rectilinear trajectories on the *xy *monitor screen plane, simulating user-generated input signals. Linear trajectories are desirable from an energy consumption point of view, as they represent the shortest path between two points. Three trajectories, one representative for each grasp, were generated using a Matlab (The MathWorks, Natick, MA, USA) script, joining the open hand position - whose coordinates are (0, 799) - to target positions (or consecutive target positions for the precision grasp, cf. bold lines in Figure 6a). In each case the trajectory crossed areas with increasing tendon tension summation (as identified by the graphs in Figure 5), while reaching the final target point and grasping the prototypical object. In practice, starting from the relaxed posture, the hand grasped the objects (that were manually handled by a human operator).

In order to simulate EMG user-generated control trajectories, i.e. a more realistic condition, trials have been conducted also using noisy input signals. White noise with different amplitudes (a maximum of 50, 70 and 100 pixels added to both *x *and *y *position signals) was generated with Matlab and added to the linear trajectories described above (see for example Figure 6b).

Further trials have been performed imagining "worst-case" user-generated trajectories, i.e. moving along "right angle" trajectories (i.e. horizontal and vertical line segments), joining the initial rest position with the identified stable points (Figure 6a, thin lines).

All trajectories have been stored in text files and used by the C program to continuously drive the robotic hand (new posture sent every 100 ms). Each time a target point was reached (circular markers in Figure 6a), the program was paused for about 2 seconds (thus stopping new positions sending).

The pre-calculated trajectories have been used to grasp the three prototypical objects held out by an operator to the robotic hand. During the experiments the hand was bind to its support platform and neither a prosthetic arm nor any wrist DoFs were implied. Thus, there was no way to perform any reaching movement towards the object, which was held out by a human operator in the artificial hand palm/fingers proximity, where we expected the CyberHand to be able to grasp it. The object was kept still and wasn't released by the operator until the robotic fingers closed and the CyberHand sustained it by itself. Twenty one trials for each grasp type have been done, for a total amount of 63 grasp trials. Position and tendon tension signals were acquired during the grasps and stored for data analysis.

The objective of this experimental setup was to understand if the "inverse-PCA" algorithm, using the specifically-built PCs matrix, practically works when coupled with an underactuated anthropomorphic hand. To this aim, *xy *trajectories both with different levels of noise - simulating the user-generated input signals - and ideally linear have been used to drive the hand. Visible factors like the tendon tensions summation trend during the grasp have been considered for qualitatively assessing the grasp and evaluate the hand behaviour in the considered conditions. The final objective of this work, indeed, is to develop a prosthesis easily controllable by an amputee and not a robotic manipulator for which many restricted precision requirements exist.

## Results

Three objects, whose shapes represent most daily used grasp types, have been grasped 21 times each using pre-calculated trajectories with different levels of added noise, for a total amount of 63 trials. The experiments showed that the hand, using such control strategy, was able to achieve stable grasps thanks to the PCs matrix specifically calculated for the CyberHand. An analysis on how tensions vary in the three considered prototypical cases, using the automatic ideal, noisy and "right-angle" trajectories, has been performed and is here presented. Graphs showing tensions variations and pictures illustrating the hand behaviour have been reported only for the more interesting precision grasp case. Nevertheless, from here forth results obtained also while performing power and lateral grasps in the considered different conditions are described and commented.

**Figure 7:**
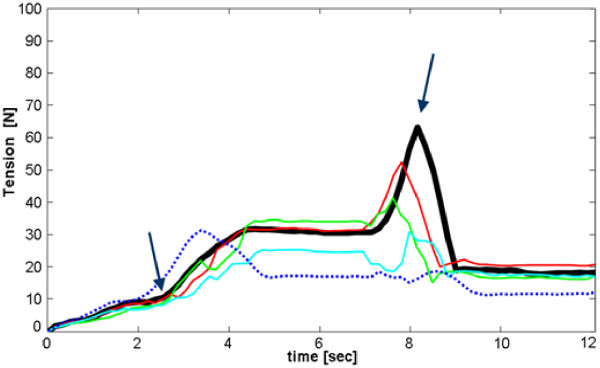
**Tendons tension trend during precision grasp**. Precision grasp using the CyberHand PCs matrix. Thumb, index and middle tendon tensions summation trend is represented while following ideal and noisy trajectories. The thick black line refers to the ideal piecewise linear trajectory in Figure 6a (bold solid line); thinner coloured curves refer to noisy trajectories (noise maximum amplitude is 50 pixels for the red curve, 70 pixels for the green curve and 100 pixels for the cyan one). The dotted curve refers instead to the "right-angle" trajectory, and has been rescaled in time to fit inside the graph. Arrows highlight the instants  when contact with the object is achieved and then lost. Tensions are calibrated in Newton using sensors characteristics.

Generally speaking, as expected the recorded tension reaches a plateau every time the trajectory is kept constant in time (that is when a stable point has been reached), but with some delay with respect to the motor pattern generation, and shows a slight overshoot before settling. This last behaviour (also noticeable in Figure 7) is caused by an high proportional constant (*K*_*P*_) in the PID algorithm, purposely set in the embedded controller in order to highlight such events.

For what concerns power grasp, the interpretation of the 5 fingers tensions summation curve is almost immediate: tension globally rises while the hand closes, until reaching a stable posture (constant tension pleateau).

The lateral grasp instead involves most of all thumb, which opposes to the volar aspect of the index: when the grasp force is sufficient, the object can be held between the thumb and index fingers. Thumb ab/adduction plays a role in influencing the thumb tension trend in time, causing tension oscillations; while the thumb is pressing against the object, an ab/adduction movement establishes a different thumb posture, with a consequent variation of its tendon tension.

In tripod/precision grasps, only thumb index and middle fingers are involved and especially the first one exerts the most of grip force, opposing to the other two fingers.

**Figure 8:**
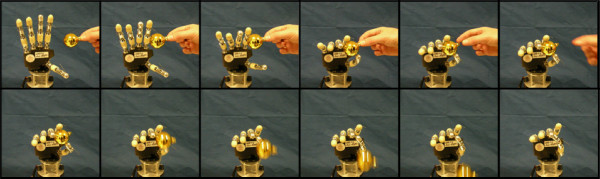
**Precision grasp video sequence**. Frame sequence showing the hand while performing a precision grasp with a spherical object. The object is firstly held by the hand, but as fingers close more and more the sphere slips away and contact is lost due to roll-back phenomenon.

Figure 7 shows characteristic curves obtained during a typical precision grasp using predefined trajectories, but the salient features they highlight (here discussed) may be generalized for all the trials performed and for different trajectories in the same grasp-area (cf. Figure 5). Tensions summation (thick black line) steadily raises once the sphere comes in contact with the fingers (first arrow); then it is followed by a plateau, when a stable grasp of the object is achieved and maintained for almost 2 seconds. Since the object is spherical and has a smooth surface, as the motors close much more the fingers get tighter: instead of reaching a second stable point (plateau), the contact is lost, the sphere slips away due to roll-back phenomenon [51] and tension sudden decreases (second arrow). A video sequence showing the slippage occurrence, caused by roll-back phenomenon, is presented in Figure 8. In the trial here described, the slip point occurs at a relatively high tendon tension summation value (about 60 N): this provides evidence for the existence of a significant stability area also for the more difficultly achievable precision grasps.

The described behaviours are obtained when the hand is controlled by ideal linear trajectories in the monitor screen reference system.

These same observations can be made when adding noise to the trajectories, with different noise gains (a displacement of 50 or 70 or 100 pixels at most). Obviously, the hand ability to firmly grasp the objects worsens while increasing noise amplitude. In all cases, a stable grasp is in the end achieved, even if with some delay and many more tension oscillations with respect to the ideal case (see for example the coloured curves in Figure 7, concerning precision grasp).

Stable grasps are obtained with some more difficulty when using "right-angle" trajectories to drive the CyberHand motion. The hand behaviour remains almost unchanged only during power grasps. On the other hand, following such a path doesn't allow to correctly perform lateral grasps any more. Firm precision grasps are obtained at lower tension values with respect to the first trials (Figure 7, dotted curve, first plateau). For this reason, when the hand is made to close more and more, the spherical object slips away almost immediately after the stable grasp point has been reached, justifying the absence of the tension peak at ~8 seconds on the dotted curve in Figure 7 (which is instead well visible on the solid curves in the same figure).

## Discussion

In carrying out the trials, the objective was to assess whether the PCA-based control algorithm is successful in driving an underactuated hand, like the CyberHand, during the most typical grasps in ADLs [48], and this issue is here discussed. Moreover, we aimed at understanding if the purposely created CyberHand PCs map works well.

The PCs matrix, resulting from postural data collected directly from the CyberHand, allows to obtain stable grasps. Despite its reduced dexterity if compared to the human hand, the robotic limb moves almost like the simulated virtual hand previously presented by the authors in [40]. Postures modulate in a gradual manner in the two-dimensional PCs space (Figure 4); i.e., fingers move without colliding, while switching between grasp areas. This map is not subject-dependent and completely fits the CyberHand, reflecting its mechanism dynamic and adaptive features. Driving the artificial hand with its own PCs map makes it able to hold objects firmly; moreover, the precision grasps area is rather wide, easily reachable and almost overlapped to the power grasps region (Figure 4 and 5). This latter feature best reflects the adaptive mechanism behaviour: the hand moulds itself in order to perform a cylindrical grasp and conforms to the object it is grasping; with the same PCs combination, if the object is small and only the thumb, index, and middle fingertips are involved, a precision grasp is achieved; if instead all fingers wrap around the object, a power grasp is obtained. In both cases, the CyberHand PCs matrix allows a well-performed and stable enclosure of the object inside the hand fingers and palm.

In order to perform a first approximation assessment of the PCA-based algorithm feasibility when dealing with the control of a real robotic hand, Santello's PCs matrix [26] was first of all used to drive the CyberHand. The artificial limb (even if not able to perform any ab/adduction movements) resulted to be almost correctly drivable also with the map resulting from a human hand dataset. A significant difference has been observed in the hand behaviour when driven with Santello's and our map. The performed trials revealed that the former facilitates lateral grasp-like hand configurations but makes the hand not capable to perfectly wrap around objects, being the thumb not completely adduced. Moreover, the hand is not able to bring fingertips close enough to steadily grip small objects in precision grasps. Drawbacks are due to the application of a human hand based mapping onto an underactuated system, which mechanically only approximates the natural hand (joints rotation axes placement, phalanxes length, etc.) but is actually unable to perform all its complex manipulative movements.

When using the new CyberHand PCs map, the first two PCs better represent the most common grasping positions, accounting for more than 90% of data variance. Grips are more stable and characterized by well defined hand joints configurations, probably only to the detriment of a less gradual overall hand motion which can be observed while varying the input signal into the *In*_*1*_, *In*_*2 *_space (cf. eq. (1)). When using the CyberHand map to perform the three considered grasps following the ideal linear trajectories, tension data show only very small fluctuations (e.g. Figure 7, thick black line). Each time all the necessary fingers are involved in grasping the object; even in precision grasps, both thumb, index and middle fingers correctly play a significant role.

Further trials have demonstrated the feasibility of our approach also in the presence of noisy inputs, used to simulate a more real working condition (i.e. myoelectric control). Even if adding random noise varying into the range between 0 and 100 pixels (which is almost high, if we consider the screen dimensions) to the original *x *and *y *position signals, the hand is able to perform the three prototypical grasps considered. Things change when moving along cathets in "right-angle" trajectories; following such a path, the hand movements appear to much less gradually vary, especially when an abrupt change from the horizontal to the vertical direction occurs. Precision grasps are far less firm and much more difficultly achievable; moreover, the hand is no more able to correctly perform lateral grasps.

These results show that not only the hand target point in the two inputs space influences grasp feasibility and stability, but also the trajectory followed in order to reach it and obviously the presence/absence of significant noise over the inputs. Linear diagonal trajectories are to be preferred to "right-angle" ones since they allow to operate a more balanced mixture between the contributions of the first input signal (*In*_*1*_, related to fingers flexion/extension) and the second input (*In*_*2*_, coupled to thumb ab/adduction movements).

## Conclusions

In this paper a control algorithm based on PCA is proposed for driving an underactuated prosthetic hand with 16 DoFs and 6 DoMs. The objective of this work has been to verify such a control strategy feasibility in different conditions, that is when driving the hand with ideal, noisy and "worst-case" user-dependant control inputs.

Similarly to what Santello did in his experiments on human hand postures [26], a new PCs matrix was obtained directly collecting a data-set of the CyberHand fingers positions from its motor encoders. In this case, the resulting two first PCs accounted for more than 90% of the variance of motion. Thus, the PCs matrix was used to drive the hand by means of a simple 2 channel (DoFs) input signal, by just "inverting" the PCA algorithm and coupling these first two PCs with the mouse cursor *x *and *y *positions. Three objects based on Iberall's and Arbib's grasp taxonomy [43] were then chosen to perform several grasping trials (power, precision and lateral grasps) and to verify whether this method could be applied to a real anthropomorphic, underactuated robotic hand.

The hand postural behaviour (Figure 4) with respect to the two inputs variation was evaluated during several grasping trials. This analysis allowed to identify where the two input signals result into a power, a precision or a lateral grasp posture, as well as to experimentally investigate positions to grasp objects in a more stable way (i.e. stability in lateral and power grasps) and in which directions fingers tendon tension increases (Figure 5).

Results obtained driving the CyberHand with ideal linear *xy *trajectories show that it is actually able to reach, correctly grasp (in terms of involved fingers, stability and hand posture while shaping around the objects) and hold objects tightly if driven with this PCA-based algorithm. The feasibility of this approach has been demonstrated evaluating the hand performances also in a more real condition, that is in the presence of noisy input control signals. Trajectories in the inputs space (i.e. couplings of the two input signals), where abrupt changes in the predominance of one of the input signals over the other one do not occur, should preferably be followed. Otherwise, grasps are achieved with much more difficulty (sometimes grasps could even fail) and the hand performances significantly worsen.

Perspective work would firstly imply the acquisition of real efferent voluntary EMG signals picked up by surface sensors, then processed in order to extract significant intention-based features to be used as input signals. By this way, it would be possible to create an advanced, intuitive and biomimetic interface modulating PCs with EMG, thus setting up a complete 2-channel controller for a bio-inspired hand prosthesis, such as the CyberHand.

## Competing interests

CC hold shares in Prensilia Srl, the company that manufactures robotic hands as the one used in this work, under the license to Scuola Superiore Sant'Anna.

## Authors' contributions

GCM and CC have full access to the data in the study and take responsibility for the integrity of the data. Design of the CyberHand: CC and MCC. Design of the PCA based concept: ELS and GM. Study concept and design: CC, GCM and MCC. Software development, acquisition and interpretation of data: CC and GCM. Drafting of the manuscript: GCM and CC. Critical revision of the manuscript for important intellectual content: CC, MCC and GM. Study supervision: MCC and GM.

All authors have read and approved the final manuscript.

## References

[B1] Biagiotti L Lotti F Melchiorri C Vassura G Design aspects for advanced robot hands Tutorial: Towards intelligent robotic manipulation, IEEE Intl Conf on Intelligent Robots and Systems 2002

[B2] Bicchi A Hands for dexterous manipulation and robust grasping: a difficult road towards simplicity IEEE Trans Rob Aut 2000 16 6 652 662 10.1109/70.897777

[B3] Carrozza MC Cappiello G Micera S Edin BB Beccai L Cipriani C Design of a cybernetic hand for perception and action Biol Cyb 2006 95 6 629 644 10.1007/s00422-006-0124-2 PMC2779386 17149592

[B4] OttoBock HealthCare Duderstdat, DE http://www.ottobock.com

[B5] Motion Control Inc Salt Lake City, UT http://utaharm.com

[B6] Liberating Technologies Inc Holliston, MA http://www.liberatingtech.com

[B7] Carrozza MC Massa B Micera S Lazzarini R Zecca M Dario P The development of a novel prosthetic hand - ongoing research and preliminary results IEEE/ASME Trans Mechatronics 2002 7 2 108 114 10.1109/TMECH.2002.1011247

[B8] Zecca M Micera S Carrozza MC Dario P Control of multifunctional prosthetic hands by processing the electromyographic signal Crit Rev Biomed Eng 2002 30 4-6 459 485 10.1615/CritRevBiomedEng.v30.i456.80 12739757

[B9] Parker P Englehart K Hudgins B Myoelectric signal processing for control of powered limb prostheses J Electromyogr Kinesiol 2006 16 541 548 10.1016/j.jelekin.2006.08.006 17045489

[B10] Kyberd PJ Holland OE Chappel PH Smith S Tregdigo R Bagwell PJ Snaith M Marcus: a two degree of freedom hand prosthesis with hierarchical grip control IEEE Trans Rehab Eng 1995 3 1 70 76 10.1109/86.372895

[B11] Touch EMAS Ltd Edinburgh, UK http://www.touchbionics.com

[B12] Craelius W The bionic man: restoring mobility Science 2002 295 1018 1021 10.1126/science.295.5557.1018 11834819

[B13] Light CM Chappell PH Development of a lightweight and adaptable multiple-axis hand prosthesis Med Eng Phys 2002 22 679 684 10.1016/S1350-4533(01)00017-0 11334753

[B14] Massa B Roccella S Carrozza MC Dario P Design and development of an underactuated prosthetic hand Proc IEEE Intl Conf on Robotics and Automation 2002 4 3374 3379

[B15] Pons JL Rocon E Ceres R Reynaerts D Saro B Levin S Van Moorleghem W The MANUS-HAND dexterous robotic upper limb prosthesis: mechanical and manipulation aspects Autonomous Robots 2004 16 143 163 10.1023/B:AURO.0000016862.38337.f1

[B16] Shulz S Pylatiuk C Reischl M Martin L Mikut R Bretthauer G A hydraulically driven multifunctional prosthetic hand Robotica 2005 23 293 299 10.1017/S0263574704001316

[B17] Cipriani C Controzzi M Carrozza MC Objectives, criteria and methods for the design of the SmartHand transradial prosthesis Robotica 2009 10.1186/1743-0003-8-29 PMC3120755 21600048

[B18] Potratz J Yang J Abdel-Malek K Peña Pitarch E Grosland N A light weight compliant hand mechanism with high degrees of freedom ASME J Biomech Eng 2005 127 6 934 945 10.1115/1.2052805 16438230

[B19] Nightingale JM Microprocessor control of an artificial arm Journal of Microcomputer Applications 1985 8 167 173 10.1016/0745-7138(85)90015-6

[B20] Tenore VG Ramos A Fahmy A Acharya S Etienne-Cummings R Thakor NV Decoding of individuated finger movements using surface electromyography IEEE Trans Biomed Eng 2009 56 5 1427 1434 10.1109/TBME.2008.2005485 19473933

[B21] Jiang N Englehart KB Parker PA Extracting simultaneous and proportional neural control information for multiple-DOF prostheses from the surface electromyographic signal IEEE Trans Biomed Eng 2009 56 4 1070 1080 10.1109/TBME.2008.2007967 19272889

[B22] Cipriani C Zaccone F Micera S Carrozza MC On the shared control of an EMG-controlled prosthetic hand: analysis of user-prosthesis interaction IEEE Trans Robotics 2008 24 1 170 184 10.1109/TRO.2007.910708

[B23] Farry KA Walker ID Baraniuk RG Myoelectric teleoperation of a complex robotic hand IEEE Trans Rob Aut 1996 12 775 788 10.1109/70.538982

[B24] Macpherson JM Humphrey DR, Freund H-J How flexible are muscle synergies? Motor control: concepts and issues 1991 Chichester, UK: Wiley 33 47

[B25] Torres Oviedo G Ting LH Muscle synergies characterizing human postural responses J Neurophysiol 2007 98 2144 2156 10.1152/jn.01360.2006 17652413

[B26] Santello M Flanders M Soechting JF Postural hand synergies for tool use J Neurosci 1998 18 23 10105 10115 9822764 10.1523/JNEUROSCI.18-23-10105.1998 PMC6793309

[B27] Santello M Soechting JF Matching object size by controlling finger span and hand shape Somatosen Moto Res 1997 14 3 203 212 10.1080/08990229771060 9402650

[B28] Mason CR Gomez JE Ebner TJ Hand synergies during reach-to-grasp J Neurophys 2001 86 6 2896 2910 10.1152/jn.2001.86.6.2896 11731546

[B29] Braido P Zhang X Quantitative analysis of finger motion coordination in hand manipulative and gestic acts Hum Mov Sci 2004 22 6 661 678 10.1016/j.humov.2003.10.001 15063047

[B30] Todorov E Ghahramani Z Analysis of the synergies underlying complex hand manipulation Proc IEEE-EMBS Intl Conf 2004 4637 4640 10.1109/IEMBS.2004.1404285 17271341

[B31] Brown CY Asada H Inter-finger coordination and postural synergies in robot hand via mechanical implementation of principal components analysis Proc IEEE/RJS Intl Conf on Intelligent Robots and Systems 2007 2877 2882

[B32] Ciocarlie MT Clanton ST Spalding MC Allen PK Biomimetic grasp planning for cortical control of a robotic hand Proc IEEE/RJS Intl Conf on Intelligent Robots and Systems 2008 2271 2276

[B33] Ciocarlie M Goldfeder C Allen P Dimensionality reduction for hand-independent dexterous robotic grasping Proc IEEE/RJS Intl Conf on Intelligent Robots and Systems 2007 3270 3275

[B34] Ciocarlie MT Allen PK Hand posture subspaces for dexterous robotic grasping Int J Robot Res 2009 28 7 851 867 10.1177/0278364909105606

[B35] Tsoli A Jenkins OC Robot grasping for prosthetic applications Proc Intl Symposium of Robotic Research 2007

[B36] Butterfass J Grebenstein M Liu H Hirzinger G DLR-Hand II: next generation of a dextrous robot hand Proc IEEE Intl Conf on Robotics and Automation 2001 109 114

[B37] Tsoli A Jenkins OC 2D subspaces for user-driven robot grasping Robotics, Science and Systems Conference: Workshop on Robot Manipulation 2007

[B38] Rosell J Suárez R Rosales C García JA Pérez A Motion planning for high dof anthropomorphic hands Proc IEEE Intl Conf on Robotics and Automation 2009 4025 4030

[B39] Schunk GmbH & Co. KG "Schunk anthropomorphic hand" 2006 http://www.schunk.com/

[B40] Magenes G Passaglia F Secco EL A new approach of multi-d.o.f. prosthetic control Proc IEEE-EMBS Intl Conf 2008 3443 3446 10.1109/IEMBS.2008.4649946 19163449

[B41] Matrone G Cipriani C Secco EL Carrozza MC Magenes G Bio-inspired controller for a dexterous prosthetic hand based on principal components analysis Proc IEEE-EMBS Intl Conf 2009 5022 5025 10.1109/IEMBS.2009.5333826 19964659

[B42] Hirose S Connected differential mechanism and its applications Proc Intl Conf on Advanced Robotics 1985 319 326

[B43] Iberall T Arbib MA Goodale MA Schemas for the control of hand movements: an essay on cortical localization Vision and Action: The Control of Grasping 1990 Norwood, NJ: Ablex 163 180

[B44] Kamper DG Cruz EG Siegel MP Stereotypical fingertip trajectories during grasp J Neurophys 2003 90 3702 3710 10.1152/jn.00546.2003 12954607

[B45] Fujiki R Arita D Taniguchi R Real-time 3D hand shape estimation based on inverse kinematics and physical constraints Proc ICIAP 2005 3617 Springer LNCS 850 858

[B46] Cipriani C Zaccone F Stellin G Beccai L Cappiello G Carrozza MC Dario P Closed loop controller for a bio-inspired multi-fingered underactuated prosthesis Proc IEEE Intl Conf on Robotics and Automation 2006 2111 2113

[B47] Pearson K On lines and planes of closest fit to systems of points in space Phil Mag 1901 2 559 572

[B48] Sollerman C Ejeskär A Sollerman hand function test. A standardised method and its use in tetraplegic patients Scand J Plast Reconstr Surg Hand Surg 1995 29 2 167 176 10.3109/02844319509034334 7569815

[B49] Bicchi A On the closure properties of robotic grasping Int J Robot Res 1995 14 4 319 334 10.1177/027836499501400402

[B50] Cutkosky MR On grasp choice, grasp models, and the design of hands for manufacturing tasks IEEE Trans Rob Aut 1989 5 3 269 279 10.1109/70.34763

[B51] Birglen L Gosselin C Kinetostatic analisys of underactuated fingers Proc IEEE Intl Conf on Robotics and Automation 2004 211 221 10.1109/TRA.2004.824641

